# Bioprospecting the Antibiofilm and Antimicrobial Activity of Soil and Insect Gut Bacteria

**DOI:** 10.3390/molecules27062002

**Published:** 2022-03-21

**Authors:** Sofía Raffaelli, Eduardo Abreo, Nora Altier, Álvaro Vázquez, Silvana Alborés

**Affiliations:** 1 Área de Microbiología, Departamento de Biociencias, Facultad de Química, Universidad de la República, Montevideo CP 11800, Uruguay; sraffaelli@fq.edu.uy; 2 Laboratorio de Farmacognosia y Productos Naturales, Departamento de Química Orgánica, Facultad de Química, Universidad de la República, Montevideo CP 11800, Uruguay; 3 Posgrado en Química, Facultad de Química, Universidad de la República, Montevideo CP 11800, Uruguay; 4 Laboratorio de Bioproducción, Plataforma de Bioinsumos, Estación Experimental INIA Las Brujas, Instituto Nacional de Investigación Agropecuaria, Canelones CP 90200, Uruguay; eabreo@inia.org.uy (E.A.); naltier@inia.org.uy (N.A.)

**Keywords:** antibiofilm, antimicrobial, soil bacteria, genome

## Abstract

Antimicrobial resistance is a growing concern in public health and current research shows an important role for bacterial biofilms in recurrent or chronic infections. New strategies, therefore, are necessary to overcome antimicrobial resistance, through the development of new therapies that could alter or inhibit biofilm formation. In this sense, antibiofilm natural products are very promising. In this work, a bioprospection of antimicrobial and antibiofilm extracts from Uruguayan soil bacteria and insect gut bacteria was carried out. Extracts from extracellular broths were tested for their ability to inhibit planktonic cell growth and biofilm formation. Genomic analysis of *Bacillus cereus* ILBB55 was carried out. All extracts were able to inhibit the growth of, at least, one microorganism and several extracts showed MICs lower than 500 µg mL^−1^ against microorganisms of clinical relevance (*Staphylococcus aureus*, *Pseudomonas aeruginosa*, and *Enterobacter cloacae*). Among the extracts evaluated for biofilm inhibition only ILBB55, from *B. cereus*, was able to inhibit, *S. aureus* (99%) and *P. aeruginosa* (62%) biofilms. Genomic analysis of this strain showed gene clusters similar to other clusters that code for known antimicrobial compounds. Our study revealed that extracts from soil bacteria and insect gut bacteria, especially from *B. cereus* ILBB55, could be potential candidates for drug discovery to treat infectious diseases and inhibit *S. aureus* and *P. aeruginosa* biofilms.

## 1. Introduction

Antimicrobial resistance is a growing concern in public health. According to WHO reports, three of the ten main causes of death worldwide were infectious diseases, causing around 6 million deaths [1] and more than 2 million deaths in children under 5 years old per year [2]. Numerous programs, such as the ESKAPE program, are being conducted to find a solution, promoting the development of new treatments. ESKAPE microorganisms [3] *Enterococcus faecium*, *Staphylococcus aureus*, *Klebsiella pneumoniae*, *Acinetobacter baumanii*, *Pseudomonas aeruginosa*, and various species of *Enterobacter* are currently the pathogens that cause most of the hospital acquired infections in the US, which can effectively “escape” the effects of antimicrobial drugs. In addition, recent studies pose that antimicrobials in developing stages that could be commercialized—approximately one third of them—will not be enough to gain ground against rapidly appearing resistance, and very few of the new drugs actually possess novelty action mechanisms [4]. Fungal infections are also a cause of worry in public health. It is estimated that, at any given time, a quarter of the population is affected by a skin fungal infection, and that 75% of women experience vulvovaginal candidiasis at least once in their lifetime [4]. *Candida albicans* is one of the most important fungal pathogens in humans, affecting particularly the immunocompromised population. Its biofilms are resistant to conventional antifungals, as well as to the host immune defenses, which has turned them into an important health issue [5].

Current research shows an important role for bacterial biofilms in recurrent or chronic infections, including those that do not respond to antibiotics [6,7]. It is known that approximately 99% of bacteria exist as biofilms, and the NIH reports that 65% of recurrent diseases and 80% of chronic diseases in humans are produced by them [8]. In addition, microorganisms are capable of forming biofilms on medical devices, such as heart valves or catheters, representing a large part of healthcare-associated infections, and can lead to increased mortality and morbidity, higher healthcare costs, and increased length of hospital stay [9]. Resistance presented by microorganisms in a biofilm can be up to 1000 times higher than their planktonic counterparts [10,11]. Moreover, cellular and community resistance can be synergic, increasing greatly the general resistance of all microorganisms involved [12].

Traditionally, persistent infections are treated with conventional antimicrobials, using higher doses and longer time periods. However, tolerance to antimicrobials can be developed through metabolic dormancy or molecular persistence programs inside the biofilm [13]. New strategies, therefore, are necessary to overcome antimicrobial resistance through the development of new therapies that could alter biofilms and destroy their constituent bacteria [14,15]. The field of natural products with activity against biofilms has shown promising results. For example, anti-biofilm activity of plant derived extracts against the infectious pathogen *P. aeruginosa* PAO1 was recently reported [16]. Various antibiofilm molecules have been identified. These molecules act in different ways, penetrating physical barriers of the biofilm, destabilizing the matrix, inhibiting quorum sensing, inducing oxidative stress, etc. [17,18,19]. Among natural products, the production of secondary metabolites with antimicrobial activity are a common feature of bacteria. However, most of them have been assessed for their antimicrobial activity against planktonic bacteria, but not against biofilms. Among these antibiofilm metabolites, nisin as well as gallidermin can inhibit the formation of staphylococcal biofilms [20,21].

Apart from *Streptomyces* and *Pseudomonas*, *Bacillus cereus* sensu lato, and *Serratia* spp. are soil-dwelling bacteria also regarded as producers of secondary metabolites. Members of these genera, capable of colonizing a range of environments, represent a source of undisclosed secondary metabolites that needs to be explored [22,23]. Hence, the search for antimicrobial and antibiofilm compounds in isolates from these genera, obtained from different environments, is envisaged as a starting point for the discovery of novel compounds with activity against resistant pathogens and biofilms.

The aim of this work was the bioprospection of extracts from Uruguayan soil bacteria or insect gut bacteria—in particular members of *Bacillus* and *Serratia* genera—that could inhibit planktonic cell growth and biofilm formation in clinically relevant microorganisms.

## 2. Results

### 2.1. Antimicrobial Activity

Previously identified microbial strains isolated from Uruguayan soil or insect gut (Table 1) were retrieved and used to inoculate broths and obtain a concentrated extract. Thirteen extracts were analyzed for their antimicrobial activity against seven microorganisms of clinical relevance (planktonic cells). The results of minimal inhibitory concentration (MIC) are listed in Table 2.

All culture broth (CB) extracts were able to inhibit the growth of at least one microorganism in the assay conditions. Additionally, several CB extracts showed MICs lower than 500 µg mL^−1^. Taking this value into consideration, three of the CB extracts had promising activity against *Staphylococcus aureus*, six against *Pseudomonas aeruginosa*, one against *Enterobacter cloacae*, and one against *Candida albicans*. ILBB162 had a MIC lower than 200 µg mL^−1^ against *S. aureus*, *P. aeruginosa*, and *E. cloacae* and ILBB55 against *C. albicans*. MICs against *E. faecalis*, *K. pneumonia*, and *A. baumannii* were above 500 µg mL^−1^ for all extracts.

Planktonic cells of *P. aeruginosa* were susceptible to 6 out of 13 of the tested CB extracts (Table 2). Because it was the most sensitive among the five Gram-negative strains, it was selected to test CB extracts for biofilm inhibition.

### 2.2. Antibiofilm Activity

#### 2.2.1. Biofilm Formation

In order to study different types of biofilms, three strains were selected: a Gram-positive bacterium (*S. aureus*), a Gram-negative bacterium (*P. aeruginosa*), and a yeast (*C. albicans*).

**Figure 1:**
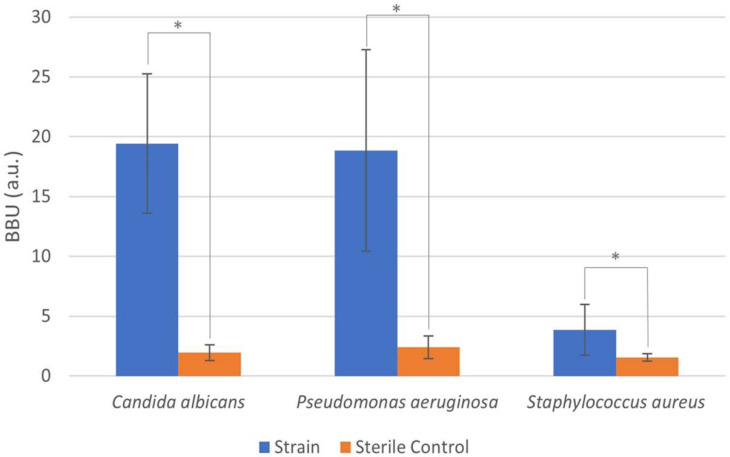
Biofilm-forming ability of *Candida albicans*, *Pseudomonas aeruginosa*, and *Staphylococcus aureus*. **Right**: Sterile control. **Left**: Strain. * *p* < 0.05.

The results of the biofilm-forming ability of the strains is shown in Figure 1, where 1 BBU (biofilm biomass unit) was arbitrarily defined as 0.1 OD_590_.

Since all the evaluated strains were classified as moderate (*S. aureus*) or strong (*P. aeruginosa* and *C. albicans*) biofilm producers, they were used in the in vitro antibiofilm activity test.

#### 2.2.2. Biofilm Inhibition Ability

The same thirteen extracts were analyzed for their ability to inhibit biofilm growth. The lowest concentration of each extract which inhibited the biofilm formation is shown in Table 3. The inhibitory concentrations ranged from 4 to 500 µg mL^−1^.

For *Pseudomonas aeruginosa* biofilms, four of the extracts could partially inhibit biofilm growth, showing statistically significant differences from the non-treated control (Table 3). In all cases, higher concentrations tested did not yield important differences in percentage of inhibition. Only the ILBB55 extract showed a relatively high inhibition with a low MIC.

**Figure 2:**
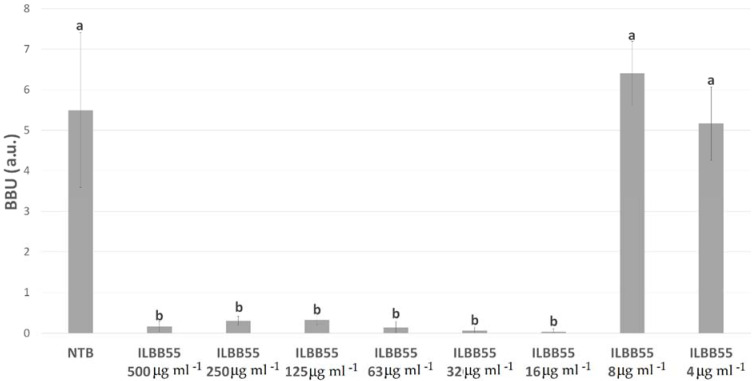
Inhibition of *Staphylococcus aureus* biofilm by different concentrations of ILBB55 extract. Different letters (a, b) represent significant differences (*p* < 0.05).

**Figure 3:**
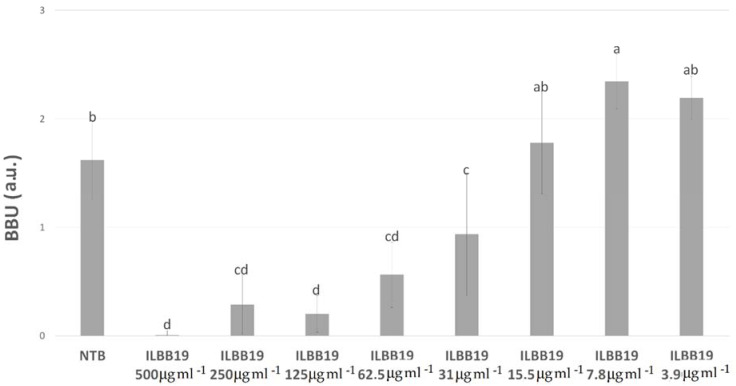
Inhibition of *Staphylococcus aureus* biofilm by different concentrations of ILBB19 extract. Different letters (a, b, c, d) represent significant differences (*p* < 0.05).

For *Staphylococcus aureus*, five extracts showed significant biofilm inhibition (Table 3). As described in Methods, minimal biofilm inhibitory concentration (MBIC) was determined as the minimal concentration able to inhibit at least 90% of the biofilm formation. Four of the extracts (ILBB19, ILBB55, ILBB145, and ILBB162) were able to inhibit at least 90% of *S. aureus* biofilm growth at some of the tested concentrations. MBICs were 500, 16, 125, and 500 µg mL^−1^, respectively. For the ILBB55 extract, the inhibition was significant at doses as low as 16 µg mL^−1^ (Figure 2). For ILBB19, the inhibition capacity diminished progressively at lower concentrations, stimulating the biofilm growth at sub-inhibitory concentrations (Figure 3).

**Figure 4:**
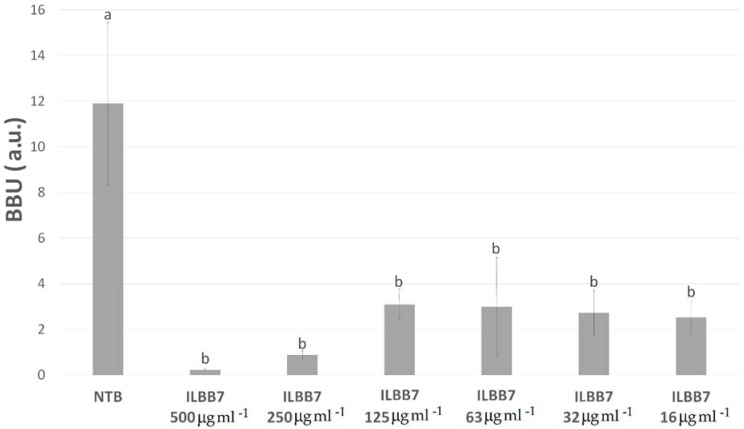
Inhibition of *Candida albicans* biofilm by different concentrations of ILBB7 extract. Different letters (a, b) represent significant differences (*p* < 0.05).

When analyzing *Candida albicans* biofilms, extracts ILBB7, ILBB55, ILBB44, and ILBB63 showed a higher inhibition percentage than the sterile broth at 16 µg mL^−1^. However, no inhibition of the *C. albicans* planktonic growth was detected at this concentration of the extracts (Table 2). Figure 4 shows that all concentrations of ILBB7 extract tested were able to inhibit *C. albicans* biofilm growth to some extent. A slight decrease in inhibition was observed at lower concentrations; however, this difference was not statistically significant.

Among the 13 extracts evaluated for biofilm inhibition, only ILBB55 was able to inhibit *C. albicans* (71%), *S. aureus* (99%), and *P. aeruginosa* (62%) biofilms.

### 2.3. Genomic Analysis of Bacillus Cereus ILB55

Since among all the extracts evaluated only ILBB55 was able to inhibit the biofilm formation of *C. albicans*, *S. aureus*, and *P. aeruginosa*, and it was also the only strain whose extract could inhibit *C. albicans* planktonic cells, this bacterial strain was selected for genomic analysis.

The assembled draft genome of *Bacillus cereus* ILBB55 contained 5,842,177 bp, 34.9% GC, and 36 contigs with parameters N50 = 573,946, L50 = 4. Genome mining carried out using antiSMASH 5.1.2 allowed us to identify types of secondary metabolite biosynthetic gene clusters that could be related to the observed antimicrobial and antibiofilm activity of ILBB55 extract.

*Bacillus cereus* ILBB55 was found to contain 16 biosynthetic gene clusters (BGC) comprising 4 clusters of non-ribosomal peptide synthetase (NRPS), 1 NRPS-like cluster, 4 ribosomally synthesized and post-translationally modified peptides (RiPPs), 1 betalactone, 1 siderophore, 1 terpene, 1 lanthipeptide class ii (RiPP), 1 sactipeptide (RiPP), 1 arylpolyene, and 1 linear azole-containing peptide (LAP, RiPP). Of these, 100% similarity with known BGC was found for the siderophore petrobactin (BGC0000942), 70% for sactipeptide thurincin H (BGC0000600), 46% for NRPS bacillibactin (BGC0000309), 40% for the betalactone (fengycin BGC0001095), and 17% with terpene molybdenum cofactor (BGC0000916).

### 2.4. Thin Layer Chromatography (TLC)

For all extracts analyzed, various phenols, organic acids, and lactones were detected by TLC. However, alkaloids, cardenolides, and indoles were not detected using this method.

## 3. Discussion

We explored extracts that could have antimicrobial and antibiofilm effects against clinically relevant microorganisms from a collection of bacteria originally obtained from complex environments where resident microbes interact and compete with other members of microbial communities [24].

*S. bockelmanii* ILBB162 and *B. cereus* ILBB55 showed the most promising activities. ILBB162 CB had a very low MIC against *S. aureus*, *P. aeruginosa*, and *E. cloacae*, whereas ILBB55 CB was the only one with a low MIC against *Candida albicans*. ILBB162 has been shown to inhibit the fungus *Pythium cryptoirregulare* through the production of diffusible and volatile metabolites in vitro [25], so this strain could be considered of biotechnological interest. Its genome mining [25] showed that it carries the BGC for althiomycin, which is known to inhibit *S. aureus*, and has the *swrA* gene coding for non-ribosomal serrawettin synthetase and can potentially produce serrawettin W2, a biosurfactant with low range antimicrobial activity that can affect the clinical *P. aeruginosa* strain PA3 [23].

CB of ILBB55 was not only able to inhibit *C. albicans* planktonic cells, but also reduced the biofilm formation of *S. aureus* and *C. albicans* at the lowest concentration of 16 µg mL^−1^. Interestingly, synthetic Dermaseptin-S1 peptide, previously isolated from *Phyllomedusa sauvagii*, inhibited *C. albicans* biofilm formation at 50 µg mL^−1^ [26]. In this work, a first chemical analysis of ILBB55 extracts was carried out by TLC, a simple and inexpensive method to determine the nature of the constituents in the extract [27]. These studies allowed the detection of organic acids and phenols, but not alkaloids, cardenolides, or indoles. Therefore, it would be interesting to encourage future works to isolate the highly active compound or mix of compounds excreted by *B. cereus* ILBB55 to evaluate the antibiofilm properties. Furthermore, we selected the strain *Bacillus cereus* ILBB55 for the genomic analysis because, as explained before, it was the only strain whose culture broth extract inhibited *C. albicans* planktonic cells and the biofilm formation of *C. albicans*, *S. aureus*, and *P. aeruginosa*. Then, genome mining allowed types of secondary metabolite biosynthetic gene clusters to be identified that could be related to the antimicrobial and antibiofilm activities. In this regard, fengycin was reported as an antifungal lipopeptide with activity against filamentous fungi but not yeast, while bacillibactin also inhibited fungal species [28]. Additionally, siderophores produced by *Bacillus* sp. GZDF3 were active against *C. albicans* [29], and it is known that iron plays a crucial role in *C. albicans* growth [30]. According to the genomic analysis, *B. cereus* ILBB55 showed a cluster 46% similar to another known cluster that codes for bacillibactin, suggesting that it could be a variant of that compound [31]. In a recent report, isolates which showed antimicrobial activity were screened for the presence of NRPS [32]. Despite most isolates being positive for NRPS, these genes were not detected for *B. cereus* isolates. However, in our work, genomic analysis of *Bacillus cereus* ILB55 showed four clusters of NRPS. This having been stated, these compounds were not expected to be present in the extracts analyzed, as their solubility in ethyl acetate is very low. Nevertheless, a global comprehension of the metabolite production of this microorganism could help in unveiling the structure of the metabolites implicated in the antibiofilm activity or aid in further investigations using other extraction methods.

Overall, results of antimicrobial assays against planktonic cells showed that all extracts inhibited the growth of at least one clinically relevant microorganism in the evaluated conditions.

Metabolites from *Bacillus pumilus* with antibacterial activity have been reported against pathogenic bacteria, including *S. aureus*, *Micrococcus luteus*, *Salmonella gallinarum*, *Pasteurella multocida*, *Salmonella enterica*, and *Escherichia coli*, with MICs of 1.6–2.0 mg mL^−1^ [33]. From our results, *S. aureus* was more sensitive to *B. pumilus* ILBB 44 and *Peribacillus butanolivorans* ILBB15 extracts, with a MIC of 0.3 mg mL^−1^. Furthermore, this extract was able to inhibit *P. aeruginosa* growth at 0.1 mg mL^−1^.

Extracellular extracts of a marine bacterial isolate, identified as *Lysinibacillus xylanilyticus*, showed that extracellular components present in the culture supernatant had antibacterial and antifungal properties [34]. We here determined that extracts from *L. xylanilyticus* ILBB63 isolated from Uruguayan soil showed a MIC of 0.6 mg mL^−1^ against two bacteria of clinical relevance, namely *S. aureus* and *E. faecalis*.

*Paenibacillus* strains isolated from various ecological niches synthesize a wide range of antibiotics, with inhibitory activity against both Gram-positive and Gram-negative bacteria. Recently, antibacterial activity against *P. aeruginosa* and *A. baumanii* but not *S. aureus* was reported for *Paenibacillus* spp. [32]. However, extracts from *Paenibacillus durus* ILBB68 isolated from soil in Uruguay were able to inhibit *S. aureus*, with a MIC of 1.25 mg mL^−1^.

Apart from antimicrobial potential against planktonic cells, extracts from different natural products have shown potential activity against microbial biofilms. Moreover, bacterial extracts from *Streptomyces chrestomyceticus* showed antimicrobial activity against *Candida* biofilms in concentrations ranging from 4 to 250 µg mL^−1^ [35], and extracts from three different species of *Bacillus* were able to inhibit *Alteromonas* sp. biofilms [36]. In this work, the antibiofilm activity of thirteen extracts from soil bacteria and insect gut bacteria is reported. The inhibitory concentrations ranged from 4 to 500 µg mL^−1^, which are very promising values compared to previous reports.

For all the extracts able to inhibit biofilm growth of *P. aeruginosa*, the minimal biofilm inhibitory concentration (MBIC) was lower than the MIC (Table 2 and Table 3). This indicates that the metabolites are interfering with the biofilm forming mechanism and not with the planktonic cell growth. Similarly, extracts ILBB7, ILBB55, ILBB44, and ILBB63 showed a significant inhibition percentage of *C. albicans* biofilm at 16 µg mL^–1^ (Table 3), but no inhibition of the planktonic growth was detected at this concentration of the extracts (Table 2). This suggests that the bacterial metabolites are able to disrupt the formation of the biofilm without affecting yeast cell growth. Mechanisms involved in this phenomenon can be very different; for example, molecules may be able to interfere with any of the steps in biofilm formation, such as adherence, cellular communication (quorum-sensing), structural integrity of the biofilm matrix, etc. Future works should be focused on the evaluation of these antibiofilm mechanisms of action.

For ILBB19, the ability to inhibit *S. aureus* biofilm diminishes progressively at lower concentrations. Interestingly, for subinhibitory concentrations, stimulation of the biofilm growth was observed (Figure 3), as previously reported by Ranieri et al. [37].

Therefore, novel microorganisms, such as soil bacteria used here, could be used as resources for the renewable production of antibiofilm compounds.

In conclusion, extracts from soil bacteria and insect gut bacteria were able to inhibit bacterial and yeast biofilm growth, showing promising results at low concentrations, being potential candidates for drug discovery to treat and prevent infectious diseases.

## 4. Materials and Methods

### 4.1. Organisms and Culture

The microbial strains used in the screening were isolated from rhizospheric soil samples, or the digestive tract of natural insect populations, and identified. Pure cultures deposited in INIA Las Brujas Bacteria Culture Collection (ILBB) were retrieved and used to inoculate broths and obtain a concentrated extract (Table 1). The bacteria were grown in 500 mL Erlenmeyer flasks containing 200 mL of modified liquid LB medium (10 g peptone, 10 g NaCl, and 0.05 g yeast extract, per litre of distilled water), incubated in a rotary shaker at 180 RPM, at 25 °C, for 48 h. Sterile broth was also used as a control in order to evaluate the influence of its components on the antimicrobial and antibiofilm activity.

### 4.2. Preparation of Extracts

Cell-free culture broths (CBs) were saturated with NaCl and filtered through cotton. A liquid–liquid extraction was then performed using ethyl acetate (AcOEt). Three portions (from three times extraction) were combined, and the solvent evaporated to dryness in a vacuum. Extracts were dissolved in dimethylsulfoxide (DMSO) for biological assays.

### 4.3. Antimicrobial Assays

#### Determination of Minimal Inhibitory Concentration (MIC)

The extracts were evaluated against the following microorganisms: *S. aureus* ATCC6538P, *E. cloacae* CCMGb3, *E. faecalis* CCMGb2, *K. pneumoniae* CCMG12716, *A. baumanii* CCMGb1, *C. albicans* ATCC 101231, and *P. aeruginosa* ATCC 15442. The MIC of the extracts was determined by the microdilution technique according to the Clinical and Laboratory Standards Institute [38]. MIC analyses were carried out in a microdilution plate with 96 wells containing Mueller–Hinton broth. The extracts were dissolved in DMSO. The initial solutions of the extracts were prepared, and further serial dilutions of the extracts and DMSO were performed. Growth controls were prepared containing inoculum and sterile broth assuring no antimicrobial effect. Gentamicin (100 µg mL^−1^) and propiconazole (125 µg mL^−1^) were used as antibacterial and antifungal controls, respectively. The MIC was determined as the lowest extract concentration (antimicrobial agent) that inhibited the visible growth of a microorganism after incubation.

### 4.4. Antibiofilm Activity

#### 4.4.1. Biofilm Formation Assay

The biofilm-forming ability of the strains was measured based on the facility of bacterial (*S. aureus* ATCC6538P, *P. aeruginosa* ATCC 15442) and yeast (*C. albicans* ATCC 101231) strains to form biofilms on solid surfaces, stained with crystal violet. The assay was adjusted to 96-well microtiter plates according to a previously published procedure [39].

Briefly, a bacterial cell suspension (1 × 10^7^ cells mL^−1^) was inoculated in sterile nutrient broth [40,41] (Oxoid), and plates were incubated at 37 °C for 24 h without shaking to allow the attachment of cells on the surface. Control wells contained only sterile nutrient broth. After incubation, non-adhered cells were removed by washing the wells with distilled water. Then, plates were dried for 40 min at 50 °C prior to crystal violet (1% *w*/*v*) staining. Adherent biofilms were washed with water to remove unbound dye. Finally, crystal violet was extracted with the bleaching solution (0.2 mL) ethanol:acetone (70:30). The optical density (OD) was determined at 590 nm using a microplate reader. The average OD of the strain was compared with average OD of the control wells (ODc), both reported as means ± standard deviations. The amount of formed biofilm was expressed as biofilm biomass unit (BBU), which was arbitrarily defined as 0.1 OD_590_ equal to 1 BBU [25]. Strains were classified according to its ability to form biofilm by following these criteria: OD ≤ ODc = no biofilm producer; ODc < OD ≤ (2 × ODc) = weak biofilm producer; (2 × ODc) < OD(4 × ODc) = moderate biofilm producer; and (4 × ODc) < OD = strong biofilm producer.

For *C. albicans*, a suspension of yeast cells (1 × 10^6^ cells mL^−1^) in potato dextrose broth (PDB, HiMedia) was incubated for 30 min in each plate well (except for the sterile control) at 37 °C. Supernatant was removed and the wells washed with PBS 1×. Then, fresh PDB was placed in the wells. Control wells containing sterile PDB were included. Plates were incubated for 24 h at 37 °C. Then, the procedure was done as described for the bacteria assay.

#### 4.4.2. Determination of Minimal Biofilm Inhibitory Concentration (MBIC)

The effect of extracts on biofilm formation was examined by using the modified microdilution method [42]. Briefly, assays against bacterial biofilms were carried out in a 96-well plate, with four replicates. Serial dilutions of the extracts and DMSO in nutrient broth were deposited, and a bacterial suspension (1 × 10^8^ cells mL^−1^) was added. Control wells containing sterile nutrient broth and nutrient broth + DMSO + bacterial suspension (non-treated biofilm, NTB) were included. The plate was incubated for 24 h at 37 °C. Supernatant was removed, and the wells were washed with distilled water and dried at 50 °C for 40 min. Then, the attached cells were dyed with 1% crystal violet solution. The crystal violet was resuspended in ethanol:acetone (70:30) and the absorbance of the solution measured at 590 nm. The absorbances of each sample, extract, NTB, and sterile nutrient broth were compared, and inhibition percentage was calculated. The MBIC was determined as the minimal concentration able to inhibit at least 90% of the biofilm formation.

For *C. albicans*, a suspension of yeast cells (1 × 10^6^ cells mL^−1^) in PDB was incubated for 30 min in each plate well (except for the sterile control) at 37 °C. Supernatant was removed and the wells washed with PBS 1×. Then, serial dilutions of the extracts and DMSO in PDB were deposited. Control wells containing sterile PDB and PDB + DMSO + yeast suspension (non-treated biofilm, NTB) were included. The plate was incubated 24 h at 37 °C. Then, the procedure was continued as described for the bacteria assay.

#### 4.4.3. Statistical Analysis

Experiments were performed at least in quadruplicate, and the averages and standard deviations were calculated for all experiments. Results were plotted as mean values with error bars that represent standard deviations. Data were statistically analyzed using ANOVA and a post hoc Tukey test for multiple comparisons (*p* value < 0.05).

### 4.5. Genomic Analysis

*Bacillus cereus* ILBB55 was used for mining biosynthetic gene clusters (BGC). For whole genome sequencing, DNA was purified with a kitDNeasy Blood & Tissue (QIAGEN, Düsseldorf, Germany) and quantified with a Qubit 4 (Thermo Fisher, Waltham, MA, USA). One gram of genomic DNA was randomly fragmented by Covaris, followed by purification by an AxyPrep Mag PCR clean upkit. The fragments were end repaired by End Repair Mix and purified afterwards. The repaired DNAs were combined with A-TailingMix, and then the Illumina adaptors were ligated to the Adenylate3′Ends DNA, followed by product purification. All kits and mixes contained BGI self-prepared reagents. The products were selected based on the insert size (350 bp). After purification, the library was qualified by the Agilent Technologies 2100 bioanalyzer and ABI Step One Plus Realtime PCR System. Finally, 150 bp paired-ends were sequenced using the Hiseq 4000 System (Illumina, San Diego, CA, USA) by BGI (Shenzhen, China). Adaptors and low-quality reads were filtered with BGI internal software SOAPnuke v1.5.2 (parameters: −l 15-q 0.2-n0.05-i). Reads were then assembled into contigs with CLC software (QIAGEN, Düsseldorf, Germany) to obtain the draft genome.

The draft genome of ILBB55 was submitted to Genbank and automatically annotated using prokka (www.ncbi.nlm.nih.gov/, accessed on 17 February 2022) as part of BioProject PRJNA745340, accession JAIQVG000000000. The version described in this paper is version JAIQVG010000000. Putative antimicrobial biosynthetic gene clusters were predicted using antiSMASH software version 5.1.2.

### 4.6. Thin Layer Chromatography (TLC)

For all extracts, TLC analyses were carried out. Extracts (10 µL) were applied in TLC plates (Silica Gel 60 F254, Merck Co., Kenilworth, NJ, USA) and developed with a variety of solvents, CH2Cl2:MeOH in different proportions (95:5 to 50:50), toluene/AcOEt/Diethylamine 70:20:10, ethyl acetate/methanol/water (100:13.5:10), and ethyl acetate/formic acid/glacial acetic acid/water (100:11:11:26) in order to analyze the metabolites contained in the extracts. Detection was carried out using different reagents and methods, such as Fast Blue B Salt, Draggendorff, Ehrlich, Van Urk’s, Kedde’s, and Natural Products II reagents for polyphenols, alkaloids, indoles, lactones, cardenolides, and phenols and phenolic acids, respectively. As general detection reagents, copper sulfate reagent/heat and UV detection at 254 and 356 nm were used [43,44,45].

## Figures and Tables

**Table 1:** Bacterial strains screened for production of antimicrobial and antibiofilm extracts.

ILBB55	*Bacillus cereus*
ILBB44	*Bacillus pumilus*
ILBB19	*Bacillus thuringiensis*
ILBB139	*Bacillus wiedmannii*
ILBB7	*Lysinibacillus fusiformis*
ILBB63	*Lysinibacillus xylanilyticus*
ILBB68	*Paenibacillus durus*
ILBB15	*Peribacillus butanolivorans*
ILB172	*Priestia megaterium*
ILBB95	*Priestia megaterium*
ILBB 162	*Serratia bockelmannii*
ILBB 219	*Serratia nevei*
ILBB 145	*Serratia ureilytica*

**Table 2:** Determination of minimal inhibitory concentration of the extracts against planktonic cells.

CB Extract	Minimal Inhibitory Concentration (µg mL^−1^)						
	*S. aureus*	*P. aeruginosa*	*E. cloacae*	*E. faecalis*	*K. pneumoniae*	*A. baumannii*	*C. albicans*
ILBB55	1250	312	2500	NI	NI	NI	125
ILBB44	312	156	2500	NI	NI	NI	NI
ILBB19	625	2500	2500	NI	NI	2500	NI
ILBB139	1250	NI	2500	2500	2500	NI	NI
ILBB7	625	625	NI	ND	NI	NI	NI
ILBB63	625	NI	2500	625	2500	NI	NI
ILBB68	1250	NI	2500	2500	2500	NI	NI
ILBB15	312	76	2500	ND	NI	NI	NI
ILBB172	625	312	2500	1250	2500	2500	NI
ILBB95	1250	1250	NI	2500	NI	2500	2500
ILBB162	156	63	76	NI	NI	1250	NI
ILBB219	1250	2500	2500	NI	NI	NI	NI
ILBB145	1250	250	1250	NI	NI	1250	NI

NI: No inhibition. ND: Not done.

**Table 3:** Inhibition of extracts on biofilm formation.

	*C. albicans*		*P. aeruginosa*		*S. aureus*	
	Concentration (µg mL^−1^)	Inhibition (%)	Concentration (µg mL^−1^)	Inhibition (%)	Concentration (µg mL^−1^)	Inhibition (%)
ILBB55	16	71	125	62	16	99
ILBB44	16	60	NI		NI	
ILBB19	125	55	NI		32	42
ILBB139	250	40	63	9	NI	
ILBB7	16	79	NI		NI	
ILBB63	16	64	500	68	NI	
ILBB68	250	68	63	13	NI	
ILBB15	16	30	NI		NI	
ILBB172	125	71	NI		NI	
ILBB95	500	93	NI		NI	
ILBB162	63	54	NI		250	77
ILBB219	32	49	NI		500	78
ILBB145	32	46	NI		125	94

NI: no inhibition.

## Data Availability

Not applicable.

## Sample Availability

Samples of the compounds are not available from the authors.

## References

[1] WHO (2018). Global Health Estimates 2016: Deaths by Cause, Age, Sex, by Country and by Region, 2000–2016.

[2] WHO (2017). Causes of child mortality. Global Health Observatory Data.

[3] Pandey R., Mishra S.K., Shrestha A. (2021). Characterisation of eskape pathogens with special reference to multidrug resistance and biofilm production in a nepalese hospital. Infect. Drug Resist..

[4] Simpkin V.L., Renwick M.J., Kelly R., Mossialos E. (2017). Incentivising innovation in antibiotic drug discovery and development: Progress, challenges and next steps. J. Antibiot..

[5] Peralta M.A., Ortega M.G., Cabrera J.L., Paraje M.G. (2018). The antioxidant activity of a prenyl flavonoid alters its antifungal toxicity on Candida albicans biofilms. Food Chem. Toxicol..

[6] Paraje M.G. (2011). Antimicrobial resistance in biofilms. Science against Microbial Pathogens: Communicating Current Research and Technological Advances.

[7] Del Pozo J.L. (2018). Biofilm-related disease. Expert Rev. Anti-Infect. Ther..

[8] Jamal M., Ahmad W., Andleeb S., Jalil F., Imran M., Asif M., Hussain T., Ali M., Rafiq M., Atif M. (2018). Bacterial biofilm and associated infections. J. Chin. Med. Assoc..

[9] Carvalho F.M., Teixeira-Santos R., Mergulhão F.J.M., Gomes L.C. (2021). The use of probiotics to fight biofilms in medical devices: A systematic review and meta-analysis. Microorganisms.

[10] Hall C.W., Mah T.F. (2017). Molecular mechanisms of biofilm-based antibiotic resistance and tolerance in pathogenic bacteria. FEMS Microbiol. Rev..

[11] Srinivasan R., Santhakumari S., Poonguzhali P., Geetha M., Dyavaiah M., Xiangmin L. (2021). Bacterial Biofilm Inhibition: A Focused Review on Recent Therapeutic Strategies for Combating the Biofilm Mediated Infections. Front. Microbiol..

[12] Penesyan A., Gillings M., Paulsen I.T. (2015). Antibiotic discovery: Combatting bacterial resistance in cells and in biofilm communities. Molecules.

[13] Jiang Y., Geng M., Bai L. (2020). Targeting biofilms therapy: Current research strategies and development hurdles. Microorganisms.

[14] Divakar S., Lama M., Asad U.K. (2019). Antibiotics versus biofilm: An emerging battleground in microbial communities. Antimicrob. Resist. Infect. Control.

[15] Estevez M.B., Raffaelli S., Mitchell S.G., Faccio R., Alborés S. (2020). Biofilm eradication using biogenic silver nanoparticles. Molecules.

[16] Alam K., Farraj D.A.A., Mah-e-Fatima S., Yameen M.A., Elshikh M.S., Alkufeidy R.M., Mustafa A.E.-Z.M.A., Bhasme P., Alshammari M.K., Alkubaisi N.A. (2020). Anti-biofilm activity of plant derived extracts against infectious pathogen-Pseudomonas aeruginosa PAO1. J. Infect. Public Health.

[17] Beoletto V.G., de las Mercedes Oliva M., Marioli J.M., Carezzano M.E., Demo M.S. (2016). Antimicrobial Natural Products against Bacterial Biofilms. Antibiotic Resistance: Mechanisms and New Antimicrobial Approaches.

[18] Song X., Xia Y., He Z., Zhang H. (2018). A Review of Natural Products with Anti-Biofilm Activity. Curr. Org. Chem..

[19] Ćirić A.D., Petrović J.D., Glamočlija J.M., Smiljković M.S., Nikolić M.M., Stojković D.S., Soković M.D. (2019). Natural products as biofilm formation antagonists and regulators of quorum sensing functions: A comprehensive review update and future trends. S. Afr. J. Bot..

[20] Shin J.M., Ateia I., Paulus J.R., Liu H., Fenno J.C., Rickard A.H., Kapila Y.L. (2015). Antimicrobial nisin acts against saliva derived multi-species biofilms without cytotoxicity to human oral cells. Front. Microbiol..

[21] Saising J., Dube L., Ziebandt A.K., Voravuthikunchai S.P., Nega M., Götz F. (2012). Activity of gallidermin on *Staphylococcus aureus* and *Staphylococcus epidermidis* biofilms. Antimicrob. Agents Chemother..

[22] Sansinenea E., Ortiz A. (2011). Secondary metabolites of soil *Bacillus* spp. Biotechnol. Lett..

[23] Clements T., Ndlovu T., Khan W. (2019). Broad-spectrum antimicrobial activity of secondary metabolites produced by *Serratia marcescens* strains. Microbiol. Res..

[24] Saxena A.K., Kumar M., Chakdar H., Anuroopa N., Bagyaraj D.J. (2020). *Bacillus* species in soil as a natural resource for plant health and nutrition. J. Appl. Microbiol..

[25] Abreo E., Valle D., González A., Altier N. (2021). Control of damping-off in tomato seedlings exerted by *Serratia* spp. strains and identification of inhibitory bacterial volatiles in vitro. Syst. Appl. Microbiol..

[26] Belmadani A., Semlali A., Rouabhia M. (2018). Dermaseptin-S1 decreases Candida albicans growth, biofilm formation and the expression of hyphal wall protein 1 and aspartic protease genes. J. Appl. Microbiol..

[27] Oshima N., Saito M., Niino M., Hiraishi Y., Ueki K., Okoshi K., Hakamatsuka T., Hada N. (2022). Using Quantitative Thin-Layer Chromatography Analysis. Molecules.

[28] Li B., Li Q., Xu Z., Zhang N., Shen Q., Zhang R. (2014). Responses of beneficial *Bacillus amyloliquefaciens* SQR9 to different soilborne fungal pathogens through the alteration of antifungal compounds production. Front. Microbiol..

[29] Sheng M., Jia H., Zhang G., Zeng L., Zhang T., Long Y., Lan J., Hu Z., Zeng Z., Wang B. (2020). Siderophore Production by Rhizosphere Biological Control Bacteria *Brevibacillus brevis* GZDF3 of *Pinellia ternata* and Its Antifungal Effects on *Candida albicans*. J. Microbiol. Biotechnol..

[30] Fourie R., Kuloyo O.O., Mochochoko B.M., Albertyn J., Pohl C.H. (2018). Iron at the centre of *Candida albicans* interactions. Front. Cell. Infect. Microbiol..

[31] Chen Z.Y., Abuduaini X., Mamat N., Yang Q.L., Wu M.J., Lin X.R., Wang R., Lin R.R., Zeng W.J., Ning H.C. (2021). Genome sequencing and functional annotation of Bacillus sp. strain BS-Z15 isolated from cotton rhizosphere soil having antagonistic activity against Verticillium dahliae. Arch. Microbiol..

[32] Tsadila C., Nikolaidis M., Dimitriou T.G., Kafantaris I., Amoutzias G.D., Pournaras S., Mossialos D. (2021). Antibacterial activity and characterization of bacteria isolated from diverse types of greek honey against nosocomial and foodborne pathogens. Appl. Sci..

[33] Chu J., Wang Y., Zhao B., Zhang X.M., Liu K., Mao L., Kalamiyets E. (2019). Isolation and identification of new antibacterial compounds from *Bacillus pumilus*. Appl. Microbiol. Biotechnol..

[34] Suvega T., Arunkumar K. (2014). Antimicrobial Activity of Bacteria Associated with Seaweeds against Plant Pathogens on Par with Bacteria Found in Seawater and Sediments. Br. Microbiol. Res. J..

[35] Singla R.K., Dubey A.K. (2019). Molecules and Metabolites from Natural Products as Inhibitors of Biofilm in *Candida* spp. pathogens. Curr. Top. Med. Chem..

[36] Viju N., Punitha S.M.J., Satheesh S. (2020). Antibiofilm activity of symbiotic Bacillus species associated with marine gastropods. Ann. Microbiol..

[37] Ranieri M.R., Whitchurch C.B., Burrows L.L. (2018). Mechanisms of biofilm stimulation by subinhibitory concentrations of antimicrobials. Curr. Opin. Microbiol..

[38] CLSI (2015). Methods for dilution antimicrobial susceptibility tests for bacteria that grow aerobically. Approved Standard.

[39] Marioni J., da Silva M.A., Cabrera J.L., Montoya S.C.N., Paraje M.G. (2016). The anthraquinones rubiadin and its 1-methyl ether isolated from Heterophyllaea pustulata reduces Candida tropicalis biofilms formation. Phytomedicine.

[40] Chebbi A., Elshikh M., Haque F., Ahmed S., Dobbin S., Marchant R., Sayadi S., Chamkha M., Banat I.M. (2017). Rhamnolipids from Pseudomonas aeruginosa strain W10; as antibiofilm/antibiofouling products for metal protection. J. Basic Microbiol..

[41] Silva S.S., Carvalho J.W.P., Aires C.P., Nitschke M. (2017). Disruption of *Staphylococcus aureus* biofilms using rhamnolipid biosurfactants. J. Dairy Sci..

[42] Teanpaisan R., Kawsud P., Pahumunto N., Puripattanavong J. (2016). Screening for antibacterial and antibiofilm activity in Thai medicinal plant extracts against oral microorganisms. J. Tradit. Complement. Med..

[43] Jork H., Funk W., Fisher W., Wimmer H. (1990). Thin Layer Chromatography Reagents and Detection Methods, Vol 1a: VCH, Weinheim, 1990 (ISBN 3-527-27834-6). xv + 464 pp. Price DM 148.00. Anal. Chim. Acta.

[44] Galand N., Pothier J., Viel C. (2002). Plant drug analysis by planar chromatography. J. Chromatogr. Sci..

[45] Pascual M.E., Carretero M.E., Slowing K.V., Villar A. (2002). Simplified screening by TLC of plant drugs. Pharm. Biol..

